# Missing Journals

**Published:** 1895-03

**Authors:** W. Horace Hoskins

**Affiliations:** Business Manager. 3452 Ludlow Street, Philadelphia


### EDITORIAL.

### Important Notice.

The Journal wishes to give notice to all of its subscribers, that, beginning with the March number, the publishers will refuse to replace any numbers lost in transmission through the mails, unless a claim is filed for the same within thirty days from the date of mailing from the publication office. Beginning with the April number the Journal will be regularly sent out between the 5th and 10th of each month. We do this because it is next to impossible to trace up Journals lost for two or three months and to place the proper responsibility for non-delivery.

W. Horace Hoskins,
Business Manager.

3452 Ludlow Street, Philadelphia.

